# Loop-Mediated Isothermal Amplification as Point-of-Care Diagnosis for Neglected Parasitic Infections

**DOI:** 10.3390/ijms21217981

**Published:** 2020-10-28

**Authors:** Catalina Avendaño, Manuel Alfonso Patarroyo

**Affiliations:** 1 Animal Science Faculty, Universidad de Ciencias Aplicadas y Ambientales (U.D.C.A.), Bogotá 111166, Colombia; cavendano@udca.edu.co; 2 Molecular Biology and Immunology Department, Fundación Instituto de Inmunología de Colombia (FIDIC), Bogotá 111321, Colombia; 3 Basic Sciences Department, School of Medicine and Health Sciences, Universidad del Rosario, Bogotá 112111, Colombia

**Keywords:** neglected parasitic infection, diagnostic test, LAMP, sensitivity

## Abstract

The World Health Organisation (WHO) has placed twenty diseases into a group known as neglected tropical diseases (NTDs), twelve of them being parasitic diseases: Chagas’ disease, cysticercosis/taeniasis, echinococcosis, food-borne trematodiasis, human African trypanosomiasis (sleeping sickness), leishmaniasis, lymphatic filariasis, onchocerciasis (river blindness), schistosomiasis, soil-transmitted helminthiasis (ascariasis, hookworm, trichuriasis), guinea-worm and scabies. Such diseases affect millions of people in developing countries where one of the main problems concerning the control of these diseases is diagnosis-based due to the most affected areas usually being far from laboratories having suitable infrastructure and/or being equipped with sophisticated equipment. Advances have been made during the last two decades regarding standardising and introducing techniques enabling diagnoses to be made in remote places, i.e., the loop-mediated isothermal amplification (LAMP) technique. This technique’s advantages include being able to perform it using simple equipment, diagnosis made directly in the field, low cost of each test and the technique’s high specificity. Using this technique could thus contribute toward neglected parasite infection (NPI) control and eradication programmes. This review describes the advances made to date regarding LAMP tests, as it has been found that even though several studies have been conducted concerning most NPI, information is scarce for others.

## 1. Introduction

Neglected tropical diseases (NTD) can usually be taken as an indicator of extreme poverty. Although this rarely happens, NTD can occur in non-endemic areas, mainly in immigrants and travellers. The most prevalent NTDs causing higher morbidity and mortality include schistosomiasis, lymphatic filariasis, onchocerciasis, trachoma and soil-transmitted helminths; these chronic infections affect at least one billion people, with many more at risk [1].

NTDs are grouped into parasitic, viral and bacterial infections in Africa, Asia and America [2]. The World Health Organisation (WHO) has identified twenty NTDs (Buruli ulcer, Chagas’ disease, cysticercosis/taeniasis, dengue fever, dracunculiasis (guinea worm disease), echinococcosis, food-borne trematodiasis, human African trypanosomiasis (HAT) (sleeping sickness), leishmaniasis, leprosy, lymphatic filariasis, onchocerciasis (river blindness), rabies, schistosomiasis, soil-transmitted helminthiasis (STH) (ascariasis, hookworm, trichuriasis), trachoma, and yaws) [3]. Eleven of these are considered the most imperative, i.e., Chagas’ disease, food-borne trematodiasis, HAT, leishmaniasis, leprosy, lymphatic filariasis, onchocerciasis schistosomiasis, STH and trachoma [2].

One of the main problems concerning NTD is that parasites once considered endemically stable are now moving; for example, it has been reported recently that Schistosoma larvae are infecting humans in Europe. Seasonal migration, extreme weather and disasters and the changes known as the “Great Acceleration” produced by the population’s rapid growth, urbanisation, habitat loss and fragmentation and loss of diversity have led to population movement across borders [4]. Climate change and global warming are increasing the probability and dissemination of many vector-transmitted diseases, including malaria, dengue, Chagas’ disease, leishmaniasis, filariasis, onchocerciasis, schistosomiasis and trypanosomiasis [3].

The 70th World Health Assembly (WHA) adopted a resolution in 2017 regarding a Global Vector Control Response for 2017–2030, whose objective was to prevent, detect, report and respond to outbreaks of vector-transmitted diseases worldwide via an integrated and exhaustive approach [3]. Preventative chemotherapy and transmission control are the two main NTD intervention methods [2]. Great advances have been made during the last decade regarding chemotherapy; however, the same cannot be said about diagnostic tools such as guidelines for chemotherapy and surveillance, mostly due to the lack of a commercially viable market for NTD diagnosis [5].

Diagnostic tests are necessary for monitoring and certifying neglected parasitic infection (NPI) elimination (except for Guinea worm having unmistakable clinical characteristics) [5]. The lack of clear diagnostic strategies has limited obtaining disease surveillance data; diagnostic tools having sufficient sensitivity and specificity for detecting infection levels which could lead to transmission are thus an essential requirement for eliminating or eradicating NPI [5]. Nucleic acid amplification is one of the most valuable tools in all fields of the life sciences, including its use orientated towards fields such as clinical medicine where the diagnosis of infectious diseases, genetic disorders and genetic traits can benefit from using this technique [6].

Polymerase chain reaction (PCR) has been extensively developed for amplifying genes since the beginning of the 1980s. PCR involves extracting nucleic acids from samples, amplification and gene detection; these steps require expensive tools, equipment and installations, thereby hampering test performance [7]. An amplification method known as loop-mediated isothermal amplification (LAMP) reaction has been developed for overcoming such limitations; this test combines speed, simplicity, high specificity and high sensitivity since LAMP’s detection limit reaches up to few copies of DNA compared to PCR. It requires less specialised equipment and infrastructure [6,7,8].

Based on the above, this review describes the state of the art concerning LAMP, a technique which has been developed for facilitating diagnosis in the field of parasites which the WHO has classified within the NTD group (except for Guinea-worm whose characteristics rule out the use of this technique). The information is presented through tables showing the genes used for designing the primers, samples used for standardising the tests and their detection limit (sensitivity).

## 2. Loop-Mediated Isothermal Amplification (LAMP)

The fundamental principle of the LAMP method developed by Notomi et al., and patented by Eiken Chemical Co., Ltd. [8], concerns the production of a large amount of DNA amplification products having a mutually complementary sequence and alternate and repeated structure. Four specific primers are used for amplification, recognising a total of six distinct sequences on target DNA: a pair of internal primers (FIP (forward inner primer), BIP (backward inner primer)) and a pair of external ones (F3, B3). The internal primers provide amplification having much greater specificity than that observed using other methods [7]. All four primers are used during the reaction’s initial stages and then just the internal primers are used for strand displacement DNA synthesis [6]. Amplification is carried out at 60–65 °C for 1 hour; nevertheless, amplification speed can be accelerated by using two Loop primers (LF (loop forward) and LB (loop backward)) [8]. This reduces the time needed for increasing and reducing incubation temperature, finishing faster than general PCR as thermal cycles are not required, thereby enabling simple isothermal equipment to be used, i.e., heat block and water bath [8].

The amplified products can be visualised by agarose gel electrophoresis or detecting turbidity based on magnesium pyrophosphate precipitation, which can be detected by the naked eye or detecting fluorescence by incorporating calcein AM dye which, accompanied by pyrophosphate ion production, triggers a reaction visible by ultraviolet light [8]. The reaction between magnesium ions and calcein AM involves a change in colour from orange to green, which can be seen by the naked eye in natural light. SYBR green I can be used instead of calcein AM. Calcein-based end-point detection is used more frequently due to its great sensitivity and notable change in colour for naked-eye observation [9].

In addition to calcein, colorimetric detection has also been done with other metal ion indicators, e.g., hydroxy naphthol blue which changes positive sample colour, ranging from violet (purple) to sky blue (blue) [10,11]. Eriochrome Black T combined with magnesium ions produces violet; however, when pyrophosphates are released during the reaction, the magnesium ions become combined with the pyrophosphates, changing colour from violet to sky blue [12]. As violet and sky blue belong to the same range of colours this hampers detecting changes by the naked eye, especially when a sample contains a small amount of the sequence to be amplified. Acid chrome blue K has been tested recently; this produces a change from red to blue if the reaction exceeds the detection limit [13]. Incorporating these indicators into the mix reduces the possibility of aerosol contamination.

Visual detection has also been tested with pH sensitive dyes, e.g., neutral red, phenol red, cresol red and m-Cresol purple colour changes, such as red to yellow (phenol red, cresol red), faint orange to pink (neutral red) or purple to yellow (m-Cresol purple). These dyes function when reaction pH begins at 8.8 and rapidly changes to a lower pH (2–3 pH unit drop) [14].

Along with SYBR green I, malachite green dye has been used as a DNA intercalator where reactions range from colourless (negative) to light blue (positive); such colour change can be visualised with the naked eye and (as it does not interfere with Bst DNA polymerase for DNA synthesis) it can be incorporated into the mix before incubation [15].

Results have also been detected by tracking phosphate ions where (by incorporating thermostable inorganic pyrophosphatase) pyrophosphate (LAMP by-product) is hydrolysed into phosphate ions, which mix and react with acidic molybdate and potassium antimonyl tartrate to form yellow ammonium phosphomolybdate trihydrate. Following ascorbic acid incorporation, the yellow compound becomes reduced to dark molybdenum blue. Negative reactions remain colourless; this option has been tested by using a closed disposable device reducing aerosol contamination due to the large amount of DNA that is produced during the reaction [16].

As analysing results by the naked eye can be somewhat subjective, amplified products can be visualised in the field by using lateral flow tests; these are cheap, easy to use, simple, rapid, light and portable. Lateral flow dipstick tests and lateral flow biosensors have been developed [9]. Lateral flow dipstick tests detect biotin-labelled cDNA that has been hybridised with FITC-labelled DNA [17], whereas lateral flow biosensors are based on nanoparticles [18]. Lateral flow tests have been seen to be superior to other monitoring techniques (colorimetric indicator, gel electrophoresis and turbidity) due to the ease of operating them and their rapid results [9,18].

A fluorometer is required for detecting fluorescence, hampering detecting results by the naked eye; however, portable isothermal fluorometers are already being marketed to enable working in the field, e.g., ESEQuant TS2 (Qiagen) [8], AmpliFire (Agdia) and Genie II (OptiGene). Devices have also been developed enabling images to be captured using smartphones. The detection technique, involving quenching unincorporated amplification signal reporters, together with a smartphone’s image analysis app, enables higher precision detection than that obtained when analysing conventional image intensity. This technique does not require opening sample tubes thereby reducing the chance of cross-contamination by aerosols. The smartphone app detects different fluorophores (FAM, HEX, ROX and CY5) [19]. Work has also been done with fluorescence microplate readers based on optical fibre bundles integrated into a smartphone. This device is compatible with a wide range of wavelengths, enabling the detection of different dyes [20]. The same principle of fluorescence has been used recently for testing a portable smartphone-based instrument for LAMP assay end-point fluorescence detection on a microfluid chip [21].

Tests have also been performed by treating samples with detergents, such as Triton-X or Sodium dodecyl sulfate (SDS), to improve sensitivity to lyse cells and release DNA into solution [22,23,24].

Flinders Technology Associates (FTA) cards (Whatman) have been studied/used for sample preparation as a direct sampling tool. Sensitivity results have been comparable to those obtained using purified DNA [25,26]. Extraction techniques using commercial kits and heat-treating samples (boil-and-spin or direct boil) have also been tested. The increased sensitivity associated with heat treatment could be due to faster DNA strand separation resulting in a more efficient LAMP test. Using heat-treated samples without compromising sensitivity eliminates the need for DNA extraction and further shortens the time to having results [27] also reducing the cost of and need for extensive laboratory infrastructure [25].

## 3. Chagas’ Disease (American Trypanosomiasis)

Chagas’ disease is caused by *Trypanosoma cruzi* and has been considered, “the most neglected of the neglected diseases.” There are gaps regarding research in this field and the development of diagnosis and treatment approaches/techniques [28]. The parasite’s stages in a mammalian host include trypomastigotes (in the blood stream) and amastigotes (intracellular replicative stage), whilst the stages in the vector (triatomine) include epimastigotes and infective metacyclic trypomastigotes [29]. *T. cruzi* is found in twenty-one continental Latin American countries from the south of the USA to the north of Argentina and Chile [30]. Although this disease has traditionally been considered endemic in South America’s tropical and sub-tropical areas (due to the migration of infected people to non-endemic countries), it has now become a global concern [28].

The infection passes from an acute to a chronic phase; the symptoms are variable and many may become spontaneously decreased during the acute phase. Appropriate treatment may lead to eradicating the parasite. Around 70% of seropositive people are asymptomatic during the chronic phase, 30% may develop cardiac or digestive problems which can last several years or decades and 2–3% have severe forms which can evolve to sudden death [28]. This parasite is known as a silent killer as chronic fatal heart disease-related consequences occur many years after patients have shown acute symptoms [29].

The parasite can be detected during the acute phase using conventional parasitological techniques such as microscopic observation of thick smears or microhaematocrite, xenodiagnosis and haemoculture as currently used diagnostic strategies are stage-dependent. However, such methods have variable sensitivity since they are operator-dependent, xenodiagnosis and haemoculture are cumbersome and delivering the results could take several weeks [31]. Serological tests are usually used for making a diagnosis since parasitaemia is intermittent and low during the chronic phase; however, the WHO has recommended that at least two tests detecting different antigens should be performed regarding *T. cruzi*’s antigenic variability; test results must coincide for a conclusive diagnosis [31,32].

The sensitivity of existing *T. cruzi* diagnostic methods regarding early diagnosis of the disease still needs to be improved; tools incorporating nucleic acid amplification thus become a good option. Molecular amplification by conventional PCR or quantitative PCR (qPCR) has been shown to have greater sensitivity and specificity than classical parasitological techniques [32], although requiring costly equipment and installations. Advances have been made regarding standardising techniques requiring less sophisticated equipment and installations in attempts to overcome such limitations, i.e., LAMP. At the time of writing this review, few biomarkers had been tested for LAMP (Table 1; Table 2), even though its sensitivity had been better than that obtained by other techniques (including PCR amplification), having provided good results regarding samples from naturally-infected patients. Diagnosing Chagas’ disease continues being a priority and needs to be improved.

The 18S rRNA sequence/marker has been the most used in attempts at standardising a LAMP which would be useful for *T. cruzi* diagnosis; however, even being the same authors, primer sets reported differ. Given the identity (83%) between *T. cruzi* and *Leishmania* spp., primers previously used for standardising a LAMP for *Leishmania* spp. have been tested for *T. cruzi*; nevertheless, *Leishmania braziliensis* and *Trypanosoma rangeli* were also detected due to the high identity when evaluating the assay’s specificity, which is why Rivero et al. [34] recommended paying special attention to false positives in those geographical regions in which these parasites prevail. One of the standardised techniques [31] in the material revised for this review involved using a prototype; the primers used were not shown in the publication. This prototype amplified a region different to 18S rRNA, being a repetitive satellite DNA sequence whose analytical sensitive limit was similar to and even greater than the sensitivity shown by qPCR (10^−2^ fg extracted DNA: 10^−1^ fg spiked blood). This further highlighted the recommendation that samples should be taken in EDTA rather than with heparin, since analytical sensitivity is greater than 0.1 parasite equivalents/mL, rising to detect 0.01 parasite equivalents/mL. Reviewing analytical sensitivity in the pertinent publications, it became apparent that the primers and technique conditions giving the greatest sensitivity were those used by Besuschio et al. [31] who demonstrated that using a commercial kit for extracting the samples had the same sensitivity as boiling and spinning them, thereby reducing sample costs and processing times.

## 4. Echinococcosis

Echinococcosis is a zoonotic parasitic disease which can be caused by larval cestodes from the family Taenidae. There are two forms, cystic (hydatidosis) caused by *Echinococcus granulosus sensu lato* and alveolar echinococcosis caused by *E. multilocularis* [37]. Echinococcosis is endemic in South America, Western Europe, Russia, the Middle East and China. Its prevalence in America ranges from 20 to 95%. The factors increasing its incidence include agriculture-based subsistence, poor socio-economic conditions, regional climate and unhygienic animal slaughtering practices [38]. The parasite requires two mammalian hosts to complete its life-cycle. The adult parasite is found in canids and the larval stage (hydatid cyst) is located in the viscera of ungulate (hooved) animals, especially sheep, goats and cattle [39]. Dogs are responsible for transmitting the disease to humans who act as incidental host; transmission between humans has not been reported to date [40].

Its clinical presentation is variable; the disease can remain asymptomatic until the cysts burst or until the amount of cysts creates pressure in the tissues surrounding them, depending on the size, location and amount of cysts [39]. The cysts may be present in any visceral organ, including the kidneys, lungs and/or brain; however, they tend to be located in the liver in 66% of cases due to the venous system of intestinal drainage [40]. Diagnosis is based on case history, clinical findings, serology and imaging. Diagnosis is confirmed by microscope observation of the cysts. An immune response is frequently undetectable as it depends on cyst location and stage, thereby hampering serological diagnosis due to its poor sensitivity [38].

Epidemiological surveillance of definitive and intermediate hosts is necessary for controlling and eradicating the disease. This involves using immunological methods such as enzyme-linked immunosorbent assay for coproantigen detection (coproELISA) and PCR. However, coproELISA can give false positives due to crossed reactions with other *Taenia* infections (including *T. hydatigena* or *T. ovis*) [39] whereas conventional PCR and quantitative real-time PCR (qRT-PCR) require sophisticated, expensive equipment, as well as infrastructure which cannot always be found in developing countries [41].

Biomarkers have been used recently for standardising LAMP to overcome such difficulties (Table 3). Standardised LAMP test sensitivity has been significant so far, even surpassing that of conventional PCR. It should be stressed that such tests can be made in remote rural areas using only a water bath or heat block and that the results can be interpreted by the naked eye [41]. Molecular diagnosis is useful for screening in the field and detecting DNA from eggs in environmental samples as an important step in identifying high-risk contaminated areas and establishing/ascertaining the routes for human infection, thereby enabling control programme progress to be determined [37].

The articles found and referenced here were all written by different research groups; most of them coincided in using the subunit 1 or 5 of the mitochondrial NADH dehydrogenase sequence. Regarding the *nad1* gene, authors have used B3 and BIP common primers, each one having an additional nucleotide at the 5′ or 3′ extreme of the sequence, whilst all the primers used for *nad5* were different. Differences regarding the units in which the authors expressed analytical sensitivity limit results hampered comparison; nevertheless, it can be stated that greater sensitivity could be obtained (10 fg) with the primers used by Ahmed et al. [41] for amplifying the *nad1* gene; depending on the *Echinococcus* species being analysed, this sensitivity can be matched also when amplifying a *cox1* fragment (10–100 fg) with the primers selected by Avila et al. [42].

## 5. Foodborne Trematodiases

Foodborne trematode infections cause more than two million disability-adjusted life years (DALY) or potential years of life lost (PYLL) worldwide every year, causing chronic liver and lung diseases. The WHO has identified four foodborne trematode/fluke genera: Clonorchis, Opisthorchis, Fasciola and Paragonimus [47]. At least fifty-six million people worldwide suffer one or more foodborne trematodiases. These parasites become acquired by eating aquatic plants (e.g., *Fasciola hepatica*), crustaceans (e.g., *Paragonimus* spp.) or fish (e.g., *Clonorchis sinensis*) [48]. These parasites’ transmission is restricted to areas where the first and second intermediate hosts coexist and where humans consume raw or pickled food or undercooked fish and other aquatic products [49].

Clonorchiasis or Chinese liver fluke disease is caused by the trematode *C. sinensis.* It was estimated in 2016 that more than fifteen million people worldwide were infected and more than two hundred million people were at risk of contracting the infection [50]. This disease is endemic in East Asian countries (China, Korea, Vietnam, Thailand) and far eastern regions of Russia [51]. This parasite’s life-cycle involves freshwater snails (the first intermediate hosts), freshwater fish and occasionally shrimps (the second intermediate hosts) and human or carnivorous mammals (the definitive hosts) [50]. Humans become infected when they consume undercooked or raw fish or shrimp, at the same time ingesting metacercariae. People infected with *C. sinensis* have a 4.47-fold greater chance of developing cholangiocarcinoma than a healthy person [52]. It is thus classified as a group biocarcinogen by the International Agency of Cancer Research (IACR) [51].

Opisthorchiasis is a disease caused by the trematode *Opisthorchis viverrini* or Southeast Asian liver fluke. It has been estimated that around ten million people are infected in the Lower Mekong Basin (Thailand, Lao PDR, Cambodia, Myanmar and Southern Vietnam) [53]. The first host is a freshwater snail whilst the second host involves several cyprinid fish species [54]. Felines are the most important reservoirs, especially in endemic areas, being responsible for persistence in the environment [53]. The main risk factor concerns consuming raw or undercooked cyprinid fish containing metacercariae [53]. The incidence of cholangiocarcinoma in people over thirty-five years old in Thailand (93.8–317.6 per 100,000 people per year) is the highest in the world, especially in the north-west where the disease is endemic. This disease is thus also classified as a group 1 biocarcinogen by the IACR [54].

Fascioliasis is a zoonotic disease caused by the trematode *Fasciola hepatica*, also known as the common liver fluke or sheep liver fluke. This parasite is the most widely distributed trematode worldwide, having been reported in around eighty-one countries [55]. This difficult-to-control disease affects a wide range of mammals, mainly ruminants, having a significant economic impact, especially in developing countries. Fascioliasis has been associated with food consumption in humans; it has been estimated that more than seventeen million people are infected and 90–170 million people live in endemic areas [56]. *F. hepatica* infection causes hepatomegaly, thickening and dilatation of the bile duct and gall bladder which can lead to cholangitis, cholecystitis and obstruction of the bile duct [57].

Migration of the parasite’s juvenile form can also cause damage to and destruction of the liver parenchyma. *F. hepatica* has been reported on five continents, its prevalence ranging from 0.19% to 100% in sheep and 0.12% to 91% in cattle. Such prevalence causes economic losses, whether arising from liver fibrosis, cirrhosis and/or cancer or reduced fertility or milk production, such losses amounting to 3.2 billion dollars per year according to worldwide calculations [57].

Paragonimiasis or lung fluke disease is caused by trematodes from the genus Paragonimus. It is caused by consuming improperly cooked freshwater crustaceans, mostly crabs or crayfish, or eating paratenic hosts. More than fifty Paragonimus species have been identified around the world in tropical and subtropical regions, mainly in Asia. Around one million people become infected every year according to WHO data [58] and close to twenty million people in Asia, the Americas and Africa could be exposed to or at risk of contracting the disease; however, its prevalence is not known exactly [59]. The infection’s prevalence depends on the abundance of the infection in animals and the prevalence of infectious metacercariae in crustacean hosts [60].

Its life-cycle requires at least two intermediate hosts as well as the definitive one; its definitive hosts include several mammals, particularly felines, canines and humans, in which it lives in pairs within a fibrous cyst in the lungs, causing pleurisy and pneumonia/bronchitis [58]. The parasite’s erratic migration pattern means that it could invade the brain or spinal cord, causing symptoms of meningitis and even host death. This disease is considered to be debilitating, as it causes morbidity and can delay socio-economic development [59].

The standard diagnostic test for these trematodes is based on microscope detection of eggs in faeces, bile, duodenal fluids or sputum (regarding *Paragonimus* spp.); however, the morphological similarities of these parasites’ eggs sometimes hampers specific diagnosis [49]. Immunodiagnostic tests (ELISA) have thus been developed for detecting trematode eggs; however, many of them cannot differentiate between past and on-going infection. LAMP tests have thus been standardised for overcoming such difficulty by detecting the parasite in samples from different hosts (Table 4 and Table 5). However, few tests have been developed to date, meaning that further efforts are needed for developing techniques which will facilitate specific diagnosis to enable successful treatment and which can contribute towards controlling and preventing these infections.

Primers amplifying the IGS and ITS2 sequences have been used for standardising LAMP for *F. hepatica* diagnosis. The same primers for IGS were used in both publications; however, the units in which analytical sensitivity limit were measured differed, thereby hindering comparison. Even though the protocol used was different, the result in both cases had good sensitivity: 1 egg [62] or 10^−5^ ng [65]. The technique´s analytical sensitivity limit when using IGS sequences was better than when using ITS2 sequences. Concerning *O. viverrine*, Le et al. [67] obtained the greatest sensitivity when amplifying the *nad1* gene, proving it to be 100 times more sensitive than PCR. Better sensitivity was apparently obtained when amplifying the ITS2 sequence when comparing two studies published concerning *P. westermani*; however, the samples used in both studies were different, meaning that more tests are needed to firmly state which set of primers and protocol provides a better sensitivity.

## 6. Human African Trypanosomiasis (Sleeping Sickness)

Human African trypanosomiasis (HAT), also known as sleeping sickness, is transmitted by the tsetse fly (Diptera: genus *Glossina*) and is found exclusively in intertropical Africa. The disease can be caused by two *Trypanosoma brucei* subspecies: *Trypanosoma brucei gambiense* and *Trypanosoma brucei rhodesiense*; each one is transmitted by a different *Glossina* subspecies [72]. *T. brucei gambiense* is the most prevalent subspecies; it is anthoponotic and endemic in central and western Africa whereas *T. brucei rhodesiense* is zoonotic and endemic in the south and east of Africa, sporadically affecting humans [73].

The amount of new chronic cases of HAT caused by *T. b. gambiense* reported from 1999 to 2018 fell from 27,862 to 953, representing a 97% reduction. The amount of new acute HAT cases caused by *T. b. rhodesiense* reported during the same period became reduced by 96%, falling from 619 to 24 cases [74]. The WHO’s goal for 2020 was to eliminate HAT as a public health problem; its indicator was fixed as being the presentation of less than 2000 cases for 2020 [73]. This objective has already been achieved, giving rise to a new objective for 2030, i.e., the total elimination of *T. brucei gambiense* transmission to achieve 0 cases [75].

As treatment is complex and potentially toxic, especially during the late phase, all efforts must be concentrated on obtaining an accurate diagnosis by the demonstration of trypanosomes in any body fluid, usually in the blood and lymph system. Once diagnosis has been made, the second step is to establish the stage of the infection in cerebrospinal fluid (CSF); however, identifying trypanosomes in CSF is not very sensitive and tests for measuring IgM, which are more sensitive, are not widely available. New biomarkers are being studied to improve diagnosis [72]. Much research has been done for standardising molecular diagnosis (Table 6 and Table 7); however, as HAT diagnosis is a speciality and the techniques are not common, technical assistance is required and reference/benchmark tests are/must be reviewed by two WHO Collaborating Centres for HAT [76].

It was difficult to compare published protocols regarding *T. brucei gambiense* since all three publications used different primers and one of them expressed sensitivity in a different unit of measurement; however, it can be stated that the sensitivity reported by Nikolskaia et al. [11] (≥1 fg) when amplifying the 5.8S ribosomal RNA-ITS2 sequence and that by Njiru et al. [86] (~1 trypanosome/mL) amplifying the TgSGP gene was extremely good. The technique was evaluated with clinical samples by amplifying the RIME sequence; Mitashi et al. [82] tested a commercial kit´s sensitivity and specificity (Loopamp *Trypanosoma brucei* kit (Eiken Chemical Co LTD) whilst test sensitivity was greater in a study by Mitashi et al. [82] (87.3% (80.9–91.8% 95% CI) in the first run and 93.0% (87.5–96.1% 95%CI) in the second run than in a study by Mugasa et al. [81] (76.9% (64.8–86.5% 95% CI)), contrary to that found regarding specificity: 92.8% (86.4–96.3% 95% CI) in the first run and 96.4% (91.1–98.6% 95% CI) in the second run and 100% (91.6–100% 95% CI), respectively.

The *T. brucei rhodesiense* SRA sequence was the most studied. Grab et al. [23] reported the greatest analytical sensitivity; they amplified a longer sequence and called it PSEUDO-SRA. Such modifications to primers made the analytical sensitivity limit when working with extracted DNA rise from 0.1–1.0 pg (1–10 parasites), when amplifying the SRA sequence, to 0.1 fg (0.001 parasite) or less. Incorporating Triton X-100 (0.5% final concentration) into a CSF sample represented a 100 to 1000-fold increase in PSEUDO-SRA LAMP assay analytical sensitivity.

The RIME sequence has been used by the authors [78,79] for screening and identifying *T. brucei* complex and differentiating *T. brucei rhodesiense* from *T. brucei brucei* and *T. brucei gambiense.* RIME and SRA positive samples were considered *T. brucei rhodesiense*, whilst if they were SRA negative, they could have been *T. brucei brucei* or *T. brucei gambiense*. LAMP sensitivity for this sequence was much greater than that for sensitivity by PCR (0.1 to 1000 trypanosomes/mL) [27]. 

Standardised protocols have been introduced for determining bovine trypanosomosis prevalence [78,79], finding that LAMP can detect more cases than microscope-detected ones, rising from 2 to 28% or from 9.3 to 41.9% depending on the region being studied.

## 7. Leishmaniasis

Leishmania is an intracellular parasite which infects phagocytic cells; it is transmitted by more than thirty species of phlebotomine sand-flies. The disease is endemic in ninety-eight countries in which it has been estimated that around 350 million people are susceptible to the disease [91]. Around 900,000 to 1.3 million new cases and 20,000–30,000 deaths occur annually in endemic areas every year [92]. The disease’s dynamics are complex and variable, depending on environmental conditions, the vector’s distribution and biology, the reservoirs involved and economic and social aspects affecting human hosts [93].

The disease’s phenotypes involve visceral (VL), cutaneous (CL) and mucocutaneous (MCL) forms. *Leishmania major* and *Leishmania tropica* cause CL in the Middle East and central Asia and the *Leishmania braziliensis* and *Leishmania mexicana* complexes in the Americas. VL is caused by the *Leishmania infantum*, *Leishmania chagasi* and *Leishmania donovani* complexes. MCL is a mucocutaneous, chronic and severe infection [91]. *Leishmania*’s transmission dynamics are complex and variable, depending on environmental conditions, the vector’s distribution and biology, the reservoirs involved and sanitary, social and economic aspects affecting human hosts [93]. Leishmania control has been based on preventing sand-fly bites, eliminating animal reservoirs (if it is zoonotic) and the early detection and effective treatment of cases in humans in the absence of an effective vaccine [93]. A key difference between VL and CL concerns *Leishmania* VL-causing species’ tendency to cause systemic disease by invading macrophages in the liver, spleen and bone marrow whilst causing minimum damage to the skin [94].

Diagnostic tools for leishmaniasis can currently be divided into three groups (parasitological, serological and molecular), each having its own advantages and disadvantages [25]. Existing tests must be improved as a means of predicting and avoiding relapses, thereby enabling treatment success to be evaluated. This requires the development of specific tests which can do more than just assessing patients’ clinical recovery, not detecting parasites and confirming cure. Diagnostic tests must thus provide immediate, truthful/accurate and confirmatory diagnosis without requiring a central laboratory [93]. LAMP technique-related biomarkers have been studied which has enabled detecting minimal amounts of parasite (1 parasite/mL) (Table 8 and Table 9) at low cost [25], thereby facilitating the identification of asymptomatic individuals and monitoring symptomatic patients’ treatment.

The 18S ribosomal sequence has been most studied when standardising LAMP for *Leishmania* spp. being shown to have a detection limit of at least 0.01 parasites for LAMP [15] or 10–100 parasites/mL for RT-LAMP [116]. Testing the protocol described by Nzelu et al. [15], León et al. [100], found that LAMP sensitivity was greater (80%: 60.3–90.0 95% CI) than that by qPCR (78.0%: 64.0–88.5% 95% CI). Adams et al. [102] tested a set of primers for amplifying the kDNA sequence. Even though the detection limit was greater (0.0001 parasites/μL) than the 18S ribosomal limit (0.01–0.001 parasites/μL), primers for kDNA CL-causing *Leishmania* species could not be amplified; however, greater sensitivity (92.3%: 74.9–99.1 95% CI) and specificity (100%: 85.8–100 95% CI) were found for VL when the technique was used with complete blood, surpassing PCR specificity (91.7%: 73–99% 95% CI), but not its sensitivity (96.1%: 80.1–99.9% 95% CI). Such sensitivity was surpassed in a study by Verma et al. [103], using a set of primers designed by Takagi et al. [117] (96.9% sensitivity: 89.6–99.2% 95% CI). The authors recommended using complete blood and not buffy coat or PBMC regarding a diagnosis of VL. Good sensitivity has also been demonstrated when using bone marrow aspirate (100%: 79.6–100% 95% CI) [103].

The kDNA sequence has been the most studied regarding *L. infantum*. The sets of primers differed in two of the four articles published; the authors did not show the sets of primers in the remaining two articles. Detection test limit was 1 fg (an equivalent of about 0.1 parasites) [96,108] or 1 parasite in 1 mL blood, i.e., 0.025 parasites per reaction [110], this being greater than that found when using K26 [98], 18S ribosomal [112] or CPB sequences [113]. Ghasemian et al. [110] found 93.6% sensitivity (95% CI) and 100% specificity (95% CI) when testing the protocol using VL patients´ blood, having efficacy comparable to that of nested PCR and surpassing the sensitivity found by Adams et al. [102]. The kDNA sequence has been tested with conjunctival swabs from dogs, as well as human blood, for a diagnosis of *L. major* [108], obtaining greater specificity (97%) than that found when canine blood is used for amplifying the CPB sequence (80%: 65.2–89.5 95% CI) [113]. The kDNA sequence has also been most studied for *L. donovani*; regardless of the set of primers used, all have shown a greater detection limit (1 fg (at least 1 parasite) [117] or 0.0001 parasites/μL [102]) than that determined for the 18S ribosomal RNA (30 pg [112]), the detection limit being comparable to the qPCR detection limit. 82.6% sensitivity and 100% specificity have been found when testing primers with skin samples; the authors [106] modified the original protocol for these studies [117].

The 18S ribosomal RNA sequence has been most studied for diagnosing CL (*L. major* and *L. tropica*), detection limits ranging from 0.01 parasites/μL (~1 fg) [107] to 160 fg [112] for *L. major* and 80 fg–3.6 pg [112] for *L. tropica*. Assays have been made for determining the usefulness of FTA cards, using previously described primers [15]; it was found that the detection limit did not become altered [107], this set of primers having the highest detection limit (0.01 parasites/μL).

## 8. Lymphatic Filariasis

Lymphatic filariasis, commonly known as elephantiasis, is a disease caused by three parasite species: *Wuchereria bancrofti*, *Brugia malayi* and *Brugia timori* [119]; *W. bancrofti* is responsible for around 90% of reported cases [120]. The disease is caused by the bite of mosquitos infected by filarial nematode larvae [121]. The disease is endemic in 73 countries and affects more than 120 million people around the world; Lourens and Ferrell have stated that around 1.1 billion people are at risk of becoming infected worldwide [121]. Infected people may be asymptomatic or even suffer serve incapacity; infection severity and intensity are determined by the duration of exposure to mosquito bites, the amount of accumulated adult larvae in the lymphatic vessels, a person’s particular immune response and the amount of secondary bacterial and fungal infections [122]. Acute infection is characterised by fever, chills, lymphadenopathy, myalgia and snoring due to microfilaria in the lungs; chronic infection is characterised by more localised symptoms such as pitting lymphedema and hydrocele, especially in the lower limbs [122].

The Global Programme to Eliminate Lymphatic Filariasis (GPELF) was established in 2000 after WHO resolution WHA 50.29 had been adopted at its 1997 assembly (along with five other infectious diseases, as being eradicable or potentially eradicable). The objective was to eliminate the disease by 2020 by interrupting the disease’s transmission and dissemination by mass drug administration (MDA), managing morbidity and preventing disabilities [119,120,121,123,124].

Considering that only a third of infected patients develop symptoms [122], the availability of serological tests for detecting infection in humans and vector xenomonitoring enormously facilitates monitoring and evaluating programmes in endemic countries; however, using xenomonitoring in programmatic mode poses a challenge since (staff/operator) suitable experience and installations for performing the molecular assays must be available [124]. This becomes more difficult if one takes into account the WHO’s recommendation that evaluating molecular xenomonitoring and sampling mosquitos should focus on individual villages (or a group of villages when target villages are small) [125]. The LAMP technique represents one way of achieving this. Studies have been carried out using different genes and ascertaining the test’s detection limit. The technique’s sensitivity has been tested using human blood and mosquito samples (Table 10 and Table 11); the results have highlighted the technique’s potential usefulness for eradicating the disease.

The nuclear scaffold/matrix attachment region sequence has been most used for *W. bancrofti*; however, Poole et al. [126] designed primers for the WbLDR sequence, improving the detection limit, passing from 1/1000th microfilaria [130] to 1/5000th microfilaria. The protocols were shown to have a detection limit equal to that of PCR [130] and qPCR [126]. Using the same protocol described by Takagi et al. [130], Kouassi et al. [131] found a larger amount of mosquitos positive by LAMP (1.8% of *Anopheles gambiae* and 0.31% of *Culex* spp.) than PCR (0% and 0.19%, respectively). The Hha I sequence has been used for standardising LAMP for diagnosing *B. malayi*, having a 1/100th microfilaria (1 pg) detection limit [126]. The protocol was used by Poole et al. [129] in a portable non-instrumented nucleic acid amplification (NINA) device, providing a stable heat source for LAMP, conserving its detection limit and having sensitivity equal to that of PCR.

## 9. Onchocerciasis (River Blindness)

Onchocerciasis, also known as river blindness, is a parasitic infection caused by *Onchocerca volvulus*. This filarial nematode is transmitted by black fly (*Simulium* spp.) bite [132]. The immunological reaction produced by the death of microfilaria is the mayor cause of morbidity; this is characterised by itching, skin depigmentation and lesions and visual disability which can progress to blindness [119]. It has been estimated that around 37 million people could be infected and 200 million at risk of contracting the infection [133]. The disease is endemic in sub-Saharan Africa, six Latin American countries and the Yemen [132].

Onchocerciasis eradication programmes have been focused on MDA for suppressing and eventually eliminating *O. volvulus* transmission. Children under ten years-old being exposed to the parasite is measured for demonstrating that transmission has been interrupted; current WHO guidelines state that the 95% confidence interval’s upper limit regarding this population’s prevalence of exposure cannot exceed 0.1% [134]. The follow-up test (Ov-16 ELISA) has 80% sensitivity and 97% specificity; a test having this level of specificity cannot measure 0.1%, meaning that tests having 99.9% specificity are required for achieving this [133]. LAMP techniques have thus been standardised, aimed at facilitating the detection of minimum amounts of antigen in both hosts and vectors (Table 12 and Table 13), thereby making it a useful surveillance tool; however, there have been few developments in this area.

The *O. volvulus* O-150 sequence has been studied for LAMP, having been shown to have a 0.0001 ng detection limit and to be 10-fold more sensitive than PCR. Higher sensitivity levels have been obtained using this protocol with clinical samples (skin biopsies), especially when using colorimetry (neutral red) instead of turbidimetry for taking the readings [136]. Poole et al. [129] used a NINA device when using the protocol described by Alhassan et al. [137], for identifying *O. volvulus* in black flies, having obtained the same detection limit as that for the original protocol and PCR.

## 10. Scabies

Scabies is a disease caused by the obligate parasite mite *Sarcoptes scabiei* var. *hominis*; this mite affects the skin’s stratum corneum where it induces a hypersensitivity reaction [138]. The 2016 Global Burden of Disease (GBD) study estimated that global point prevalence was around 147 million, accompanied by a 455 million case incidence annually [139]. The highest scabies rate is found in tropical regions, including East Asia, Southeast Asia, Southern Asia, Oceania and tropical Latin America [138]. This disease, one of the commonest worldwide, was incorporated into the list of NTDs by the WHO in 2017 [139].

Definitive diagnosis is mainly based on microscope observation of skin scrapings; however, scabies diagnosis cannot be ruled out just by a negative result as false negatives could occur when there are few mites [140]. ELISA tests and molecular techniques for amplifying nucleic acids (PCRs) have been developed as alternative methods [141]. Molecular methods also reduce the amount of false negatives and have enabled differentiating *S. scabiei* var. *hominis* infestation from a zoonotic infestation, e.g., *S. scabiei* var. *canis,* which cannot be differentiated via microscope and is important in terms of public health and for establishing treatment [141,142].

The difficulty involved in using PCR is that it requires specialised infrastructure and equipment. So, once again, the LAMP technique becomes a good diagnosis option for communities living in remote places and having limited access to laboratories. Unfortunately, very few approaches have been made in this field (Table 14).

In spite of the published protocol’s detection limit being stated in nanograms (i.e., when most protocols use femtograms or picograms), Fraser et al. [141] obtained 100% sensitivity (0.86–1.00 95% CI) and 92.50% specificity (0.80–0.98 95% CI) when comparing LAMP results with those for PCR.

## 11. Schistosomiasis

Schistosomiasis, also known as bilharzia, is a tropical parasitic disease causing significant morbidity and mortality rates, especially in developing countries; it is the third most reported tropical disease and the second most reported in the sub-Saharan region [143]. Several *Schistosom*a species have been described; however, most cases are attributed to three species in particular: *Schistosoma haematobium*, *Schistosoma japonicum* and *Schistosoma mansoni*. Reports of the disease being caused by species such as *Schistosoma mekongi* and *Schistosoma intercalatum* are less common [143]. *S. haematobium* and *S. mansoni* are the most prevalent species in Africa and the Middle East whereas only *S. mansoni* occurs in Latin America and *S. japonicum* in Asia [144].

It has been estimated that around 250 million people are affected by the disease worldwide and more than 779 million are at risk of becoming infected, causing close to 280,000 deaths annually [145]. This makes it one of the five zoonotic diseases whose global health rate has been underestimated [143]. These blood-feeding flukes are responsible for significant clinical complications involving the liver, intestines, spleen and bladder, leading to chronic inflammation and fibrosis [146].

Programmes must involve intensive intervention measures and efficient monitoring to ensure success regarding the disease’s prevention and elimination [146]. The difficulty in demonstrating significant reductions in infection levels in regions having very low prevalence and basal intensity is one of the challenges involved in eliminating the parasite [147]. The techniques usually used for diagnosing the disease include conventional microscope diagnosis, serological tests determining the presence of antibodies, tests for detecting parasite antigens and DNA detection methods (PCR) [146].

Molecular techniques used for DNA detection have aroused much interest considering that an ideal schistosomiasis diagnosis must be highly sensitive and specific, be easy to use and interpret, rapid, low cost, capable of using/processing different types of sample and be potentially applicable in disease-endemic areas having extremely limited economic resources. LAMP is a technique which meets all such requirements and therefore represents an alternative tool regarding other more complex molecular methods [148]. Several approaches have thus been made for standardising and using it (Table 15 and Table 16), although such tests are still not widely available.

*S. mansoni* has been the most studied *Schistosoma* species, the mitochondrial minisatellite DNA region and Sm1-7 repeat fragment sequences more than others. The mitochondrial minisatellite DNA region sequence has been shown to have the best detection limit (0.01 fg [152]) when using the primers designed by Fernández-Soto et al. [158]. The authors modified the protocol for obtaining this limit, increasing incubation time from 60 to 120 minutes; this improved analytical sensitivity from 1 fg to 0.01 fg and increasing incubation time did not affect test specificity. Using this set of primers which had already been tested in urine [152], faeces [158] and snails [155], the research group improved the protocol, transforming it into a cold maintenance dry format, suitable for potentially manufacturing as a kit for ready-to-use for schistosomiasis diagnosis [150]. Price et al. [151] amplified the Sm1-7 sequence for evaluating the effect of extraction on LAMP performance, finding greater sensitivity and specificity when samples were extracted with a LAMP-Qiagen kit (100% sensitivity: 96–100% 95% CI; 100% specificity: 59–100% 95% CI), compared to using PCR-Qiagen, PCR_LAMP-PURE, LAMP_LAMP_PURE kits.

The ITS-1 [149], IGS [153,156] and DraI [163,164] sequences have been studied for *S. haematobium* diagnosis, a greater detection limit having been obtained with DraI (0.1 fg). Gandasegui et al. [153] worked with the IGS region, finding a larger amount of samples to be positive by colorimetry than by turbidimetry (i.e., when adding SYBR Green I to the reaction); however, the effect of the extraction method has been controversial since Gandasegui et al. [153] found that sensitivity was greater when DNA was extracted with a commercial kit. Gandasegui et al. [156] found greater sensitivity when working with the Rapid-Heat LAMPellet method when the test was done from urine sample supernatant or sediment, finding more positive individuals when working with DNA from sediment.

Even though the best detection limit was not obtained for *S. mansoni* (1 pg) or *S. haematobium* (0.1 pg) with the ITS-1 region, the primers designed for amplifying this region can be used for detecting different *Schistosoma* species (*S. haematobium*, *S. intercalatum*, *S. mansoni* and *S. bovis*).

## 12. Soil-Transmitted Helminthiases (Intestinal Worms)

It has been estimated that around 2 billion people (almost a quarter of the world’s population) are infected by STHs [166], causing around 135,000 deaths per year [167]. Most of these infections are caused by roundworms (*Ascaris lumbricoides* and *Strongyloides stercoralis*), whipworms (*Trichuris trichiura*) and hookworms (*Necator americanus* or *Ancylostoma duodenale*) [168]. The WHO has included *Ascaris lumbricoides, Trichuris trichiura*, *Necator americanus* and *Ancylostoma duodenale* in its list of STH-caused NTDs [169]. Although each species has specific characteristics, the WHO groups STHs for control purposes in terms of similarity between geographically-defined endemic groups and the risk of becoming affected, treatment involving the same drugs, using the same tools for diagnosis and involving a similar mechanism having negative impact on human health [166]. These parasites are endemic in more than 100 countries [166], being more prevalent in developing regions in Africa, Asia and the Americas as well as being the most prevalent NTDs worldwide [169].

The WHO has endorsed a preventative chemotherapy strategy for controlling STH-related morbidity without needing prior individual diagnosis for groups at specific risk who are living in endemic areas, this being the most profitable approach [166]. Ab-based assays for STH diagnosis and widely-used faeces’ microscopy (although these are not standardised) may help primo-infection diagnosis and cases where microscopy has proved negative [170].

Work is currently underway regarding the development of a PCR for clinical management and public health, although the tests are still not widely available [170]. Developing techniques having high specificity will contribute towards knowledge regarding real prevalence in target regions due to the costs incurred in adapting/updating infrastructure and providing the necessary equipment for carrying out immunoassays or PCRs. It must be borne in mind that eliminating eggs from faeces is intermittent when attempting to establish the control programmes proposed in the WHO’s Guidelines: Preventive chemotherapy to control soil-transmitted helminth infections in at-risk population groups [166].

This makes the LAMP technique a good option regarding less specialised infrastructure and equipment requirements, being cheaper and a test having greater specificity [169]; however, few approaches to date have involved using this technique (Table 17). This technique’s validation and use will enable filling one of the gaps found in the WHO’s 2017 guidelines concerning its recommendations about the need for new field diagnosis for detecting and treating target groups and monitoring the impact of preventative chemotherapy on morbidity and transmission [171].

Different genes have been studied for diagnosing intestinal worms, all being able to detect at least 1 egg. Regarding *Strogyloides* spp., LAMP sensitivity (10^−3^ ng) was greater than PCR sensitivity (0.1 ng) when amplifying the 18S rRNA sequence from DNA extracted from *S. venezuelensis* [175]. No positive PCR results were obtained when using the same primers on urine samples [173]. Concerning *T. trichiura*, the set of primers which amplified the ITS-2 region designed by Ngari et al. [172] was able to detect very low levels of infection, having greater sensitivity than the Kato–Katz technique. Even though the detection limit obtained by Rashwan et al. [174] after designing primers amplifying the β-tubulin region was not better (1 pg), the advantage of this protocol lies in its ability to detect *T. trichiura*, *A. lumbricoides* and *N. americanus* eggs. Even though the Kato–Katz technique’s sensitivity and specificity are low and LAMP’s detection limit is considered good, work is still needed on optimising protocols for improving the sensitivity and specificity obtained for *T. trichiura* (77%, sensitivity, 88% specificity [172]) and for *A. lumbricoides* (96.3% sensitivity, 61.5% specificity [176]).

## 13. Taeniasis/Cysticercosis

Human taeniasis is a worldwide parasitic infection, although communities in developing countries bear the greatest burden. Three species can affect humans: *Taenia saginata* (the beef tapeworm), *Taenia solium* (the pork tapeworm) and *Taenia asiatica* (the Asian tapeworm) [178]. Neurocysticercosis (NCC) causes 29% of neurological crises [179]; it is caused by the pork tapeworm’s larval stage and should not be confused with the type of taeniasis resulting from adult tapeworm infection [180]. *T. solium* occupied first place in the global scale of food-transmitted parasites in 2014 [181]. Latin America, Africa, South East Asia, India, China and Nepal are endemic areas for NCC [180]. The WHO included NCC caused by *T. solium* in its 2012 route map for addressing NTDs, highlighting it as a neglected zoonotic disease (NZD) [181].

MDA involves treating most non-infected people, which, in this case, involves incurring high costs and treatment-associated risks, selective treatment can thus be considered an alternative. However, it involves difficulties due to diagnosis limitations related to identifying carriers since the symptoms are vague and there is a lack of availability of sensitive, specific and applicable detection methods in endemic areas as well as the costs involved in such procedure [181].

The LAMP technique would be a very useful tool for contributing towards reducing the cysticercosis rate in developing regions, except when cysticerci are calcified since there are no circulating antigens during this stage. The WHO’s Department of Control of Neglected Tropical Diseases (WHO/NTD) and the Special Programme for Research and Training in Tropical Diseases met in 2015 to discuss the difficulties regarding *T. solium* diagnosis; consensus was reached indicating that diagnosis should include the different *Taenia* species to facilitate the diagnosis and control of other infections caused by *Taenia* spp.

Likewise, there was consensus that diagnosis should focus on symptomatic patients for detecting people having viable cysts and who needed to be referred for diagnostic imaging and other procedures/management, including anti-helminth treatment. This took into account rural and remote communities where the disease is endemic and having suitable diagnostic facilities/installations [179]. The LAMP technique requires further work with symptomatic patients to demonstrate its usefulness in diagnosing them, even though its good detection limit has been highlighted and it can be carried out in the field using little specialised equipment (Table 18).

All the articles published that were related to standardising a LAMP for diagnosing *T. solium* came from the same group, which itself worked mostly with the *cox1* sequence due its ability for differentiating between the three *Taenia* species (*T. solium*, *T. saginata* and *T. asiatica*), plus greater capacity for detecting positive samples. The protocols used have been shown to be able to detect the parasite’s different stages and have greater sensitivity than multiplex PCR.

## 14. Conclusions

The authors referenced here have demonstrated greater sensitivity for the LAMP technique than PCR in almost all cases whether the DNA used in a test came from natural infections or reference strains, regardless of parasite genus or species; in the few cases in which the test did not surpass PCR detection limit, it at least equalled it. This led to all authors coinciding in concluding that LAMP is a simpler and cheaper technique since it requires less sophisticated equipment, thereby enabling the test to be carried out in conventional laboratories or even directly in the field. This makes the technique a useful tool for contributing toward surveillance and control programmes aimed at eliminating NPI.

## Figures and Tables

**Table 1:** Publications found in the US National Library of Medicine National Institutes of Health (NCBI-PubMed) reporting the standardisation of the loop-mediated isothermal amplification (LAMP) technique as a useful tool for diagnosing Chagas’ disease (American trypanosomiasis) in the definitive host.

Parasite	Marker	Extracted/Spiked/Natural DNA	Sample	Sample Size	Sensitivity	Reference
*T. cruzi*	Satellite nuclear repeat region (231 bp)	Extracted DNA	CL Brener and DM28 strains	-	CL: 5 fg DM28: 50 fg	[33]
*T. cruzi*	Repetitive satellite DNA sequence	Extracted, spiked and natural DNA	Reference strains belonging to the six Discrete Typing Units (DTUs) and human blood	33	Extracted DNA: ≥10^−2^ parasite equivalents/mL (0.3 fg) Spiked EDTA blood: 10^−2^ parasite equivalents/mL Spiked heparinised blood: 10^−1^ parasite equivalents/mL	[31]
*T. cruzi*	18 S rRNA	Spiked and natural DNA	Human blood	27	50 parasites/mL	[34]
*T. cruzi*	18 S rRNA	Spiked/natural DNA	Tulahuen strain	-	100 fg	[35]
*T. cruzi*	18 S rRNA	Spiked DNA	Tulahuen strain	-	1 fg	[36]

**Table 2:** Publications found in the US National Library of Medicine National Institutes of Health (NCBI-PubMed) reporting the standardisation of the LAMP technique as a useful tool for diagnosing Chagas’ disease (American trypanosomiasis) in the vector.

Parasite	Marker	Extracted/Spiked/Natural DNA	Sample	Sample Size	Sensitivity	Reference
*T. cruzi*	18S rRNA	Spiked/natural DNA	Triatomine bugs	52	100 fg	[35]

**Table 3:** Publications found in the US National Library of Medicine National Institutes of Health (NCBI-PubMed) reporting the standardisation of the LAMP technique as a useful tool for diagnosing echinococcosis in the definitive host.

Parasite	Gene	Extracted/spiked/natural DNA	Sample	Sample Size	Sensitivity	Reference
*E. granulosus* complex	*cox1*	All of them	Protoscoleces/dog faeces/faeces collected from the environment	Dog faeces: 5 Faeces collected from the environment: 61	10–100 fg	[42]
*E. granulosus* complex	*nad1*	Natural DNA	Hydatid cysts containing protoscoleces and associated germinal layers	100	10 fg	[41]
*E. granulosus* complex	*nad1*	Diluted protoscolex or egg lysate and natural DNA	Protoscolex or eggs	60	1/50 of a single protoscolex or egg	[43]
*E. multilocularis*	Mitochondrial *nad5*	Extracted/ natural DNA	Protoscolex tissue/dog faeces	189	1 pg	[44]
*E. granulosus*	Mitochondrial *nad5*	Extracted and natural DNA	Isolates and faecal samples from dogs	190	10 pg	[45]
*E. granulosus*	Repeat region sequence	Spiked DNA	Protoscoleces recovered from affected sheep livers	-	1 pg/200 mg faeces	[46]

**Table 4:** Publications found in the US National Library of Medicine National Institutes of Health (NCBI-PubMed) reporting the standardisation of the LAMP technique as a useful tool for diagnosing foodborne trematodiases in the definitive host.

Parasite	Gene	Extracted/Spiked/Natural DNA	Sample	Sample Size	Sensitivity	Reference
*C. sinensis*	*cox1*	All of them	Human stool samples	120	1 egg/100 mg of faeces	[61]
*F. hepatica*	IGS	Spiked DNA	Samples collected from infected dog liver and faeces	-	1 egg	[62]
*F. hepatica*	ITS2	Natural DNA	Sheep faeces	15	10^−3^ ng	[63]
*F. hepatica*	ITS2	Natural DNA	Sheep and cattle faeces	Sheep:39 Cattle: 25	10^−4^ ng	[64]
*F. hepatica*	IGS	Extracted DNA	Individual worms and eggs	Adults: 14 Eggs: 1	10^−5^ ng	[65]
*F. gigantica*						
*O. viverrini*	Microsatellite 6 (OVMS6)	Extracted DNA	Adult worms		1 ng	[66]
*O. viverrini*	*nad1*	Natural DNA	Vietnamese isolate (OvBD1) and samples collected from humans	-	10^−3^ –10^−4^ ng	[67]
*O. viverrini*	ITS1	Natural DNA	Adult worms from experimentally infected hamsters and children’s faeces	37	10^−3^ ng	[68]
*P. westermani*	Ty3/gypsy-like LTR retrotransposon (Rn1)	Natural DNA	Canine blood	124	2.7 fg	[69]
*P. westermani* *(Oriental lung fluke)*	ITS2	Natural DNA	Adult worms from experimentally-infected dogs, eggs from patients’ sputum and pleural fluid	Samples from patients’ sputum and pleural fluid: 17	10^−8^ ng	[70]

**Table 5:** Publications found in the US National Library of Medicine National Institutes of Health (NCBI-PubMed) reporting the standardisation of the LAMP technique as a useful tool for diagnosing foodborne trematodiases in the intermediate host.

Parasite	Gene	Extracted/Spiked/Natural DNA	Sample	Sample Size	Sensitivity	Reference
*C. sinensis*	*cox1*	All of them	Metacercariae from naturally-infected freshwater fish	-	100 fg of DNA	[61]
*C. sinensis*	ITS2	Natural DNA	Freshwater snails, shrimp and freshwater fish	Snails: 497 Shrimp: 42 Freshwater fish: 33	10 fg (0.0002/snail)	[71]
*F. hepatica*	IGS	Extracted DNA	Snails	Cercariae: 1	10^−5^ ng	[65]
*F. gigantica*						
*O. viverrini*	*nad1*	Natural DNA	samples collected from fish	-	10^−3^–10^−4^ ng	[67]
*P. westermani* *(Oriental lung fluke)*	ITS2	Natural DNA	Metacercariae from freshwater crabs and crayfish	Muscle samples from infected freshwater crabs or crayfish: 35	10^−8^ ng	[70]

**Table 6:** Publications found in the US National Library of Medicine National Institutes of Health (NCBI-PubMed) reporting the standardisation of the LAMP technique as a useful tool for diagnosing HAT (sleeping sickness) in the definitive host.

Parasite	Gene	Extracted/Spiked/Natural DNA	Sample	Sample Size	Sensitivity	Reference
*T. brucei brucei*	RIME	Spiked DNA	GVR35 strain	-	Blood treated with RBC lysis solution: 0.04 trypanosomes/mL	[22]
*T. brucei gambiense*	5.8S ribosomal RNA -ITS2	Extracted DNA	*T. brucei gambiense* DAL 972 isolate	-	≥1 fg	[11]
*T. brucei gambiense*	TgSGP	Spiked and natural DNA	Human blood samples	-	~1 pg (~10 trypanosomes/mL)	[77]
*T. congolense*	18S rRNA (CON2-LAMP)	Natural DNA	Cattle blood	420	ND	[78]
*T. viva* *xT. brucei rhodesiense*	Satellite DNA					
	SRA					
*T. congolense*	18S rRNA (CON2-LAMP)	Natural DNA	Cattle blood	295	ND	[79]
*T. vivax*	Satellite DNA					
*T. brucei rhodesiense*	SRA					
*T. brucei gambiense*	TgSGP	Experimentally infected DNA	Saliva and urine from vervet monkeys	6	ND	[80]
*T. brucei gambiense*	RIME	Natural DNA	Human blood	181	ND	[81]
*T. brucei gambiense*	RIME	Natural DNA	Human blood	253	ND	[82]
*T. congolense*	18S rRNA (CON2-LAMP)	Natural DNA	Canine blood	6	ND	[83]
*T. viva* *xT. brucei rhodesiense*	Satellite DNA					
	SRA					
*T. brucei rhodesiense*	RIME SRA	Natural DNA	Human blood and CSF	4	ND	[84]
*T. brucei rhodesiense*	RIME Pseudo-SRA LAMP	Extracted DNA	*T. b. rhodesiense* IL1852	-	0.1 fg genomic DNA (0.001 parasite)	[23]
	RIME	Spiked DNA	Human blood and CSF	-	Blood: 10 trypanosome/mL CSF: 10 trypanosomes/mL	
	Pseudo-SRA LAMP				Blood: 0.01 trypanosomes/mL CSF: 0.1 trypanosomes/mL	
*T. brucei*	RIME	Extracted/natural DNA	*T. b. rhodesiense* isolate LVH 56, *T. b. gambiense* isolate NW2/human blood and CSF	-	0.01 trypanosomes/mL	[85]
*T. brucei gambiense*	TgSGP	Extracted DNA	Isolate PT41 DNA	-	10 trypanosomes/mL	[86]
		Spiked/natural DNA	Mouse blood/human blood and CSF	10	~1 trypanosomes/mL	
*T. brucei rhodesiense*	Pseudo-SRA LAMP RIME	Natural DNA	Human blood	128	ND	[87]
*S*ub-genus *Trypanozoon*	RIME	Extracted and Natural DNA	Human blood and CSF	20	0.001 trypanosomes/mL	[27]
*T. brucei rhodesiense*	SRA	Experimentally infected/natural DNA	Mouse blood/human blood and CSF	Human sample:6	Purified DNA: 10 trypanosomes/mL Heat-treated buffy coat: 0.1 pg (1 trypanosome/mL) ^a^	[88]
*T. brucei* group *T. congolense*	PFR protein A	Experimentally infected/ extracted DNA	Mouse blood *T. congolense* IL-3000	-	*T. brucei* GUTat3.1: 1 pg *T. congolense*: 1 ng	[89]

^a^ using heat-treated buffy coat.

**Table 7:** Publications found in the US National Library of Medicine National Institutes of Health (NCBI-PubMed) reporting the standardisation of the LAMP technique as a useful tool for diagnosing HAT (sleeping sickness) in the vector.

Parasite	Gene	Extracted/Spiked/Natural DNA	Sample	Sample Size	Sensitivity	Reference
*T. brucei*	RIME-LAMP	Natural DNA	Tsetse flies	150	ND	[90]
*T. brucei gambiense*	TgSGP	Spiked and natural DNA	Tsetse fly	-	~1 pg (~10 trypanosomes/mL)	[77]

**Table 8:** Publications found in the US National Library of Medicine National Institutes of Health (NCBI-PubMed) reporting the standardisation of the LAMP technique as a useful tool for diagnosing leishmaniasis in the definitive host.

Parasite	Gene	Extracted/Spiked/Natural DNA	Sample	Sample Size	Sensitivity	Reference
*L. siamensis*	18S ribosomal RNA	Extracted/natural DNA	MON-324; MHOM/TH/2010/TR/human blood samples	50	10^2^ parasites/mL (0.0147 ng/μL)	[95]
*L. infantum*	kDNA	Extracted DNA	MHOM/BR/2011/COS	-	1 fg	[96]
*L. amazonensis*	kDNA	Extracted/ experimentally infected DNA	MCAN/BR/2002/BH401 strain/skin samples from hamsters	-	10 pg to 100 pg ^a^	[97]
*L. infantum*	K26	Extracted/spiked/natural DNA	MHOM/BR/74/PP75 strains/human blood samples	219	1 fg DNA 100 parasites/mL	[98]
*L. major*	CPB	Natural DNA	Human tissue material	81	20 fg	[99]
*L. tropica*					200 fg	
*Leishmania* spp.	18S rRNA	Extracted/natural DNA	Cutaneous leishmaniasis-causing strains and direct smears from CL patients’ skin lesions	50	1 × 10^−2^ equivalent parasites	[100]
*Leishmania* spp.	18S rRNA	Natural DNA	Human skin biopsies	2	1 parasites/μL	[101]
*Leishmania* spp.	18S ribosomal DNA	Extracted/natural DNA	Visceral leishmaniasis-causing strains and cutaneous leishmaniasis-causing strains/human blood samples and skin samples	CL: 105 VL:50	0.01 to 0.001 parasites/μL	[102]
	kDNA				0.0001 parasites/μL ^b^	
	Histone H3 gene				0.01 parasites/μL ^c^	
*L. donovani*	kDNA	Extracted/natural DNA	*L. donovani* AG83, *L. major* ASKH and *L. tropica* WR 683/VL: human peripheral blood or bone marrow aspirate ^d^ PKDL: tissue biopsy	VL: 66 from blood and 15 from bone marrow PKDL ^d^: 67	1 fg	[103]
*L. tropica*					1 pg	
*L. major*					100 pg	
*L. martiniquensis*	18S rRNA	Extracted/natural DNA	*L. martiniquensis* (MHOM/TH/2011/PG)	-	0.13 parasites/μL	[104]
*Leishmania* spp.	ITS-1	Extracted/natural DNA	Reference strains/human blood samples	-	*L. donovani*: 0.1 pg *L. major*, *L. aethiopica*, *L. tropica*, *L. infantum chagasi*: 1 ng	[105]
*L. donovani*	kDNA	Natural DNA	Human skin lesions	31	ND	[106]
*L. major*	18S ribosomal RNA	Parasite applied to FTA card/natural DNA	*L. major* (MHOM/SU/1973/5ASKH)/human tissue material	122	0.01 parasites/µL	[107]
*L. infantum*	kDNA	Extracted/natural DNA	*L. infantum* (MCAN/CN/90/SC)/conjunctival dog swab	111	1 fg	[108]
*L. siamensis*	18S ribosomal RNA	Spiked DNA	Human saliva and blood	-	10^3^ parasite/mL	[109]
*L. infantum*	kDNA	Natural DNA	Human blood samples	87	1 parasite/mL	[110]
*Leishmania* spp.	18S ribosomal RNA	Extracted/natural DNA	VL and CL reference strains	-	0.01 parasites	[15]
*L. major*	18S ribosomal DNA	Spiked/natural DNA	Mouse blood/swab samples	41	1 × 10^3^ parasites/mL	[111]
*L. major*	18S rRNA	Extracted DNA	*L. donovani* (MHOM/IN/1996/THAK35), *L. infantum* L4, *L. major* 230/119, *L. major* 159/06 and *L. major* LV39	-	1.6–160 fg ^e^	[112]
*L. tropica*					80 fg–3.6 pg ^e^	
*L. infantum*					46 fg–46.2 pg ^e^	
*L. donovani*					30 pg	
*L. infantum*	CPB	Extracted/natural DNA	*L. infantum* (MHOM/TN/80/IPT1)/canine blood samples	75	50 fg	[113]
*L. donovani*	kDNA	Extracted/natural DNA	*L. donovani* AG83 (MHOM/IN/83/AG83)/human blood and bone marrow samples	117	1 fg (0.01 parasites)	[114]
*L. donovani*	kDNA	Extracted/natural DNA	*L. donovani* (MHOM/IN/80/DD8)/human blood	185	1 fg	[115]
*Leishmania* spp.	18S ribosomal RNA	Extracted/natural DNA	*L. donovani* (MHOM/SD/68/IS)/human blood and skin biopsies	Blood: 30 biopsies: 17	10–100 parasites/mL	[116]
*L. donovani*	kDNA	Extracted/natural DNA	*L. donovani* strain DD8/human blood samples	10	1 fg	[117]

^a^ 10 pg of DNA using poly-acrylamide gel to visualise the result and 100 pg for colorimetric detection with SYBR Safe. ^b^ no amplification with CL-causing *Leishmania* species. ^c^ no amplification at all with *L. guyanensis* and *L. braziliensis* strains. ^d^ PKDL: post kala-azar dermal leishmaniasis. ^e^ depending on which *Leishmania* species were tested and the primer sets used.

**Table 9:** Publications found in the US National Library of Medicine National Institutes of Health (NCBI-PubMed) reporting the standardisation of the LAMP technique as a useful tool for diagnosing leishmaniasis in the vector.

Parasite	Gene	Extracted/Spiked/Natural DNA	Sample	Sample Size	Sensitivity	Reference
*Leishmania* spp.	18S rRNA	Extracted/natural DNA	Sand flies	50	1 × 10^−2^ equivalent parasites	[100]
*L. martiniquensis*	18S rRNA	Extracted/natural DNA	Sand flies	Sand flies: 380	0.13 parasites/µL or 10 promastigotes in one sand fly	[104]
*L. major*	18S rRNA	Natural DNA	Sand flies	-	10^1^–10^6^ Promastigotes/100 μL–0.1 parasites	[118]
*Leishmania* spp.	18S ribosomal RNA	Extracted/natural DNA	Sand flies	397	0.01 parasites	[15]

**Table 10:** Publications found in the US National Library of Medicine National Institutes of Health (NCBI-PubMed) reporting the standardisation of the LAMP technique as a useful tool for diagnosing lymphatic filariasis in the definitive host.

Parasite	Gene	Extracted/Spiked/Natural DNA	Sample	Sample Size	Sensitivity	Reference
*B. malayi*	*Hha* I repeat (BmHha I)	Natural DNA	Human blood samples	8	1/100th of microfilaria (1 pg)	[126]
*W. bancrofti*	WbLDR				1/5000th of microfilaria	
*W. bancrofti*	Nuclear scaffold/matrix attachment region	Natural DNA	Human blood samples	Blood: 3067	ND	[127]
*W. bancrofti*	Nuclear scaffold/matrix attachment region	Natural DNA	Human blood samples	Blood: 3067	ND	[128]
*B. malayi or B. timori*	*Hha* I repeat	Extracted/natural DNA	Feline blood samples	1	0.001 ng	[129]
*W. bancrofti*	Nuclear scaffold/matrix attachment region	Extracted/spiked DNA	Human blood	-	1/1000th of microfilaria	[130]

**Table 11:** Publications found in the US National Library of Medicine National Institutes of Health (NCBI-PubMed) reporting the standardisation of the LAMP technique as a useful tool for diagnosing lymphatic filariasis in the vector.

Parasite	Gene	Extracted/Spiked/Natural DNA	Sample	Sample Size	Sensitivity	Reference
*W. bancrofti*	Nuclear scaffold/matrix attachment region	Natural DNA	Mosquitoes	15,568	ND	[125]
*B. malayi*	*Hha* I repeat (BmHha I)	Natural DNA	Infected mosquitos	8	1/100th of microfilaria (1 pg)	[126]
*W. bancrofti*	WbLDR				1/5000th of microfilaria	
*W. bancrofti*	Nuclear scaffold/matrix attachment region	Natural DNA	Mosquitoes	Mosquitoes:14,334	ND	[131]
*W. bancrofti*	Nuclear scaffold/matrix attachment region	Natural DNA	Mosquitoes	Mosquitoes:1,260	ND	[127]
*W. bancrofti*	Nuclear scaffold/matrix attachment region	Natural DNA	Mosquitoes	Mosquitoes:16,073	ND	[128]
*W. bancrofti*	Nuclear scaffold/matrix attachment region	Extracted/spiked DNA	Mosquitoes	-	1/1000th of microfilaria	[130]

**Table 12:** Publications found in the US National Library of Medicine National Institutes of Health (NCBI-PubMed) reporting the standardisation of the LAMP technique as a useful tool for diagnosing onchocerciasis (river blindness) in the definitive host.

Parasite	Gene	Extracted/Spiked/Natural DNA	Sample	Sample Size	Sensitivity	Reference
*O. volvulus*	Mitochondrial *cox*1	Extracted/natural DNA	Skin biopsies	150	ND	[135]
*O. volvulus*	O-150	Extracted/natural DNA	Skin snips	70	0.0001 ng	[136]

**Table 13:** Publications found in the US National Library of Medicine National Institutes of Health (NCBI-PubMed) reporting the standardisation of the LAMP technique as a useful tool for diagnosing onchocerciasis (river blindness) in the vector.

Parasite	Gene	Extracted/Spiked/Natural DNA	Sample	Sample Size	Sensitivity	Reference
*O. volvulus*	*GST1a*	Extracted DNA/Experimentally infected DNA	Black flies	4	0.01 ng	[126]
*O. volvulus*	*GST1a*	Extracted/spiked DNA	Black flies	-	0.01 ng	[137]

**Table 14:** Publications found in the US National Library of Medicine National Institutes of Health (NCBI-PubMed) reporting the standardisation of the LAMP technique as a useful tool for diagnosing scabies in the definitive host.

Parasite	Gene	Extracted/Spiked/Natural DNA	Sample	Sample Size	Sensitivity	Reference
*S. scabiei*	ITS-2	Extracted/natural DNA	Mite/animal skin scrapings	64	Single mite DNA: 0.02 ng Skin scraping: 0.008 ng	[141]

**Table 15:** Publications found in the US National Library of Medicine National Institutes of Health (NCBI-PubMed) reporting the standardisation of the LAMP technique as a useful tool for diagnosing schistosomiasis in the definitive host.

Parasite	Gene	Extracted/Spiked/Natural DNA	Sample	Sample Size	Sensitivity	Reference
*S. mansoni* *S. intercalatum* *S. haematobium* *S. bovis*	ITS-1	Extracted DNA	Adult worms	-	*S. mansoni* and *S. intercalatum*: 1 pg *S. haematobium*: 0.1 pg *S. bovis*: 10 pg	[149]
*S. mansoni*	Mitochondrial minisatellite DNA region	Extracted/natural DNA	Cutaneous and hepatic biopsies and appendix biopsy	3	ND	[150]
*S. mansoni*	Sm1-7 repeat fragment	Natural DNA	Human urine and stool	111	ND	[151]
*S. mansoni*	Mitochondrial minisatellite DNA region	Spiked/natural DNA	Human urine	28	0.01 fg	[152]
*S. haematobium*	IGS	Natural DNA	Human urine	172	ND	[153]
*S. mansoni*	Sm1- 7 repeat fragment	Extracted/natural DNA	Human faeces	383	32 fg	[154]
*S. mansoni*	Mitochondrial minisatellite DNA region	Natural DNA	Human faeces	427	ND	[155]
*S. haematobium is*	IGS	Spiked/natural DNA	Egyptian strain, NR-31682/human urine samples	94	1 fg	[156]
*S. japonicum*	Retrotransposon SjR2	Natural DNA	Human serum	110	ND	[157]
*S. mansoni*	Mitochondrial minisatellite DNA	Experimentally infected DNA	Mice faeces	-	1 fg	[158]
*S. japonicum*	Highly repetitive retrotransposon (SjR2)	Natural DNA	Rabbit faeces	6	0.08 fg	[159]

**Table 16:** Publications found in the US National Library of Medicine National Institutes of Health (NCBI-PubMed) reporting the standardisation of the LAMP technique as a useful tool for diagnosing schistosomiasis in the vector.

Parasite	Gene	Extracted/Spiked/Natural DNA	Sample	Sample Size	Sensitivity	Reference
*S. japonicum*	28S ribosomal (Sj28S) gene	Natural DNA	Snail soft tissues	4006 ^a^	1/50	[160]
*S. mansoni*	Mitochondrial minisatellite DNA region	Natural DNA	Snail soft tissues	1175 ^b^	ND	[155]
*S. mansoni*	ITS	Natural DNA	Snail soft tissues	Individually: 11 Pools: 8	70 fg	[161]
*S. mansoni*	IGS 28S-18S ribosomal RNA	Natural DNA	Snail soft tissues	41	1 fg	[162]
*S. haematobium is*	Dral repeat fragment	Natural DNA	Snail soft tissues	103	0.1 fg	[163]
*S. mansoni*	Sm1-7 repeat fragment					
*S. haematobium is*	Dral repeat fragment	Natural DNA	Snail soft tissues	11	0.1 fg	[164]
*S. mansoni*	Sm1-7 repeat fragment					
*S. japonicum*	28S rDNA gene	Natural DNA	Snail soft tissues	362	100 fg	[165]

^a^ 232 pooled snail samples from single snails. ^b^ 46 pooled snail samples from single snails.

**Table 17:** Publications found in the US National Library of Medicine National Institutes of Health (NCBI-PubMed) reporting the standardisation of the LAMP technique as a useful tool for diagnosing soil-transmitted helminthiases (intestinal worms) in the definitive host.

Parasite	Gene	Extracted/Spiked/Natural DNA	Sample	Sample Size	Sensitivity	Reference
*T. trichiura*	ITS-2	Natural DNA	Human faeces	137	98.6 × 10^−9^ ng/μL or a single egg	[172]
*Strongyloides* spp.	18S rRNA	Natural DNA	Human urine	24	ND	[173]
*A. lumbricoides*	β-tubulin isotype 1	Extracted/natural/spiked DNA	Human faeces	21	1 pg or a single egg	[174]
*T. trichiura*				15		
*N. americanus*				19		
*Strongyloides* spp.	18S rRNA	Extracted/natural DNA	Rat faeces and urine Human faeces	12	10^−3^ ng	[175]
*A. lumbricoides*	ITS-1	Natural DNA	Human faeces	581	10.8 ng (equivalent to a single egg)	[176]
*N. americanus*	ITS-2	Natural DNA	Human faeces	106	0.4 fg	[177]

**Table 18:** Publications found in the US National Library of Medicine National Institutes of Health (NCBI-PubMed) reporting the standardisation of the LAMP technique as a useful tool for diagnosing taeniasis/cysticercosis in the definitive host.

Parasite	Gene	Extracted/Spiked/Natural DNA	Sample	Sample Size	Sensitivity	Reference
*T. solium*	*cox1*	Extracted/natural DNA	Human faeces	102	1 copy of target gene	[182]
*T. solium*	*cox1*	Natural DNA	Tapeworms	51	ND	[183]
*T. solium*	*cox1* and *clp*	Natural DNA	Human faeces	36	ND	[184]
*T. solium*	*cox1* and *clp*	Extracted DNA	Proglottids and cysticerci	168	1 copy of target gene	[185]

## Abbreviations

BIP	backward inner primer
CL	cutaneous leishmaniasis
*clp*	cathepsin l-like cysteine peptidase
*cox1*	cytochrome *c* oxidase subunit 1
CPB	cysteine protease B
CSF	cerebrospinal fluid
DNA	deoxyribonucleic acid
DTUs	discrete typing units
FIP	forward inner primer
FTA	Flinders Technology Associates
GBD	global burden of disease
GPELF	global programme to eliminate lymphatic filariasis
*GST1a*	glutathione s- transferase-1
HAT	human African trypanosomiasis
IGS	ribosomal intergenic spacer
IACR	International Agency of Cancer Research
ITS	internal transcribed spacer
ITS-1	internal transcribed spacer 1 region
ITS-2	the second internal transcribed spacer region
fg	femtogram
kDNA	kinetoplast DNA minicircle sequence
LAMP	loop-mediated isothermal amplification
LB	loop backward
LDR	long DNA repeat
LF	loop forward
MDA	mass drug administration
MCL	mucocutaneous leishmaniasis
*nad1*	NADH dehydrogenase subunit 1
*nad5*	NADH dehydrogenase subunit 5
NADH	nicotinamide adenine dinucleotide (NAD) + hydrogen
ND	not determined
NINA	non-instrumented nucleic acid amplification
NPI	neglected parasite infections
NTD	neglected tropical disease
NZD	neglected zoonotic disease
ng	nanogram
PCR	polymerase chain reaction
PFR	paraflagellar rod
pg	picogram
RBC	red blood cell
RIME	repetitive insertion mobile element
rDNA	ribosomal deoxyribonucleic acid
rRNA	ribosomal ribonucleic acid
SDS	Sodium dodecyl sulfate
STH	soil-transmitted helminths
SRA	human serum resistance-associated
SGP	specific glycoprotein
VL	visceral leishmaniasis
WHA	World Health Assembly
WHO	World Health Organisation
